# Illinois State Medical Society, Held at Dixon, Ill., May 17, 1870

**Published:** 1870-06

**Authors:** 


### THE ILLINOIS STATE MEDICAL SOCIETY.

### FIRST DAY—MORNING SESSION.

DIXON, ILL., May 17.

The State Medical Convention held its opening session in the court-house, of this city, on yesterday morning. The meeting was called to order at 10:30 A. M., Dr. G. W. Albin, of Mayo, First Vice-President, in the chair.

Dr. O. M. Everett, chairman of the committee of arrangements, welcomed the convention to the hospitalities of the profession and people of Dixon.

Dr. J. C. Corbus, secretary of the committee of arrangements, read the following list of delegates as present, with the proper credentials: Profs. N. S. Davis and R. N. Isham, Chicago Medical College; Drs. Chas. M. Morse and M. A. McClelland, Military Tract Medical Association; Drs. James Murphy, G. H. Johnson, and James L. Hamilton, Peoria Medical Association; Dr. David Prince, Morgan County Medical Association; Drs. R. G. Bogue and E. L. Holmes, Chicago Medical Society; D. J. Crist, Bloomington Medical Society; Drs. G. W. Phillips and G. W. Havitt, Lee County Medical Society; Dr. G. W. Albin, permanent member from Neoga; E. R. Willard, from De Witt county; R. S. Willard, from Vandalia, Fayette county; Dr. Saml. J. Jones, St. Luke's hospital, Chicago; Dr. Jas. S. Whitmire, permanent member from Metamora; Drs. E. P. Cooke and J. C. Corbus, Union Medico-Pathological Society, from La Salle and adjoining counties; Drs. Chas. Hunt and O. M. Everett, permanent members from Dixon; Dr. M. M. Robins, Fox River Valley Medical Association; Drs. G. L. Monnee and Leland, Medico-Pathological Society of La Salle and adjoining counties; Dr. Edwin Powell, of Rush Medical College; Dr. D. M. Young, Fox River Valley Medical Association; Dr. Samuel C. Plumma, Iowa and Illinois Central District Medical Association; Drs. John P. McClanahan and W. S. Sterling, of the Military Tract Medical Society; Dr. D. E. Foote, permanent member from Belvidere; Dr. D. M. Vosburg, of Earlville.

The following named gentlemen were unanimously elected as members of the State Medical Society: Dr. D. A. Sheffield, of Appleton; Dr. R. W. Shaw, of Ashton; Drs. E. P. Leavors and J. R. Felker, of Amboy; Drs. C. C. Satuner and W. Cohely, of Princeton; also, Drs. Henry E. Payne, D. W. Lowe, G. R. Bradwell, and Charles McAllister.

The reports of standing committees were then called for, and the following reports read by the chairmen of the respective committees: "Drugs and Medicines," "Ophthalmology," "Plaster of Paris in Fractures," and "Otology." They were all referred to the committee on publication.

On motion of Dr. Davis, the annual dues were fixed at $3.

The convention then adjourned till 2 P. M.

### AFTERNOON SESSION.

The meeting was called to order at 2 o'clock P. M., Dr. Albin in the chair.

Dr. Everett introduced to the convention, Col. Dement, the mayor of this city, who welcomed them in a neat and appropriate speech, and extended to them the hospitalities of the city. Dr. J. S. Whitmire read a supplementary report on practical medicine, which was referred to the committee on publication, and, as calls for other reports from standing committees were not responded to, the society resolved itself into a committee of the whole on general discussion.

The subject of scarlatina and other contagious diseases was taken up, and the rest of the afternoon devoted to its discussion.

At 6 o'clock the convention adjourned till 8 P. M.

EVENING SESSION.

The convention was called to order at 8 P. M., Dr. Albin in the chair.

The following gentlemen were elected as Permanent Secretaries: Drs. W. Walton, of Ridout, and Thos. Winston, of Foreston.

On motion of Dr. N. S. Davis, Dr. Walton and Dr. W. H. C. Donaldson were placed on the nominating committee to represent Stephenson and Whiteside counties.

The order of business for the evening was then declared to be the discussion of carbolic acid in treating suppurating wounds. Dr. N. S. Davis said it would be important to bring out facts relating to the injurious effects of this treatment, if such could be established. Dr. Young, of Aurora, called on Dr. Bogue, of Chicago, to give his views, who gave way to Prof. Isham. Dr. Isham did not express full faith in carbolic acid. In wounds which were to be cured by primary intention, carbolic acid did not answer the purpose. Where there was coagulation there must be liquefaction, before a course of convalescence could occur. Carbolic acid seemed to be useless in lacerated wounds, attended with compound fractures. Carbolic acid was sometimes very useful as an antiseptic. But another remedy was still more efficacious, namely, a solution of iodine in chloroform. This arrested the course of suppuration. The modifying influences of carbolic acid were confined to its antiseptic qualities.

Dr. Bogue said his experience had not been favorable to carbolic acid. He would never use it with a recent wound. It had no beneficial influence on flaps or stumps, and in lacerated wounds the use of carbolic acid, in his judgment, was no more beneficial than in incised wounds. This remedial agent had had an extensive trial in Chicago hospitals, and had been, in general, a failure. He simply used it as a covering over the dressing. Next to the wound its antiseptic qualities would recommend it simply as an outside covering, not that its contact with the wound would be of any benefit. Its application to stumps, in many cases, had so shriveled the flesh that granulation and cicatrization could not take place. In some cases of large suppurating cavities it might yield some advantage. As to any poisonous effects, as producing pyæmia and the like results, he could give no facts from his own experience.

Dr. Young, of Aurora, said his experience verified that of Drs. Bogue and Isham. He thought he had remarked two cases of blood poisoning, which were aggravated by the direct application of carbolic acid to the wound. In one case that of a man who had his foot crushed by a railroad accident, abscesses formed rapidly in different parts of the body, without any direct swelling of the foot. He feared that the carbolic acid had something to do in contributing to this result.

Dr. Prince, of Jacksonville, thought that the application of carbolic acid resulted unfavorably because it was used in too great strength. The point to be ascertained was, how great strength would kill the animal or vegetable parasites, and yet not injure the patient. The fact that a part shriveled under its application only proved that the solution was too strong. He used only three or four grains of the crystals to an ounce of water. The hypothesis was that suppuration was aggravated by pus. In a pure state, carbolic acid would arrest the putrefaction, and so far operate to prevent absorption of the poison into the system.

When primary intention was not sought for, but granulation, as following the ordinary organic course of suppuration in the wound, he believed carbolic acid, in a proper degree of strength, would be beneficial, as tending to destroy any vegetable spores which might have settled in the wound. He used chloride of zinc, glycerine, and carbolic acid, mixed together in nearly equal proportions, over the isinglass dressing. He believed that no remedy would have more beneficial results as thus applied. The doctor illustrated his views at considerable length, by examples from his own practice. The remedy was also beneficial, oftentimes, in obstetric practice. There was frequently a laceration of the vagina or uterus, or a lodgment of clotted blood, whose putrefaction would certainly aggravate the danger to the patient. In puerperal fever and in certain inflammations of the throat, he had found the use of carbolic acid highly gratifying in its success. He could not but be enthusiastic in favor of this remedy, as his own use of it had been so uniformly successful.

Dr. John Murphy, of Peoria, said that one of the most remarkable facts connected with the profession was the different results of the same remedies at the hands of different physicians. He regarded carbolic acid as invaluable in the case of recent wounds, in preventing suppuration, or keeping it within healthy bounds. His way of application was by means of sutures not tightly drawn, which gave ample chance for the carbolic acid lotion to be absorbed.

Dr. Isham thought that sometimes enthusiasm for a new dressing caused practitioners to lose sight of principles. He proceeded to argue that chemical decomposition was the logical result of the use of carbolic acid. In ordinary cases, where the system of the patient was healthy, it was not beneficial, but where the system of the patient was vitiated by organic disease, and suppuration in the wound was created with great rapidity, it might be possible that some benefit would be derived. He had no confidence whatever in carbolic acid in wounds which were in process of cure by primary intention.

Dr. Whitmire, of Metamora, contended, in relation to the assertion that carbolic acid was a poison, that there were no absolute poisons in nature, but only relatively so. The use of carbolic acid, as advocated by its friends, was not poisonous, but highly beneficial.

Dr. N. S. Davis thought that carbolic acid was going through the history of all valuable remedies. It had been used to excess in many cases, and was, therefore, regarded by too many now as valueless. If used with moderation and skill, he believed that it could be used to advantage. In clean wounds, through a healthy tissue, he did not believe any application was beneficial; but in the case of badly suppurated wounds he had much confidence in carbolic acid. When the violence of extreme opinions had subsided on the question of carbolic acid, he felt it would be regarded as a valuable addition to the pharmacopœia. In cases of its medicinal use internally, it had been fortunate in some cases. In a case of morbid sensitiveness of the mucous membrane of the stomach, to enable the latter to bear food, he had found it of much use. Dr. Davis proceeded to describe several instances of a highly interesting nature illustrative of its successful internal use. In cancerous diseases of his experience, he had found the judicious use of carbolic acid very important as effecting relief, and sometimes marked benefit. It was given internally, and also used as a local application; but he had not used it as an injection. He believed it to be a very potent agent in holding such malignant diseases at bay, although he could not say that he regarded it as a complete cure.

After a brief further discussion on this question, the convention adjourned till 9 A. M., to-morrow.

---

SECOND DAY—MORNING SESSION.

DIXON, Ill., May 18.

The Illinois State Medical Convention held their second day's session on this morning, opening at 9 o'clock. Dr. G. W. Albin in the chair.

The following committees were appointed for the succeeding year:

Surgery—Prof. E. Andrews, of Chicago, chairman; Drs. D. Prince, of Jacksonville, and Webster, of Metamora.

Obstetrics—Dr. DeLaskie Miller, of Chicago, chairman; Drs. John P. McClenahan, of Norwood, and O. Everts, of Dixon.

Drugs and Medicines—Prof. N. S. Davis, of Chicago, chairman; Drs. B. Griffith, of Springfield, and E. Ingalls, of Chicago.

Criminal Abortions—Dr. W. H. Byford, of Chicago, chairman; Drs. Chas. Hunt, of Dixon, and Foote, of Belvidere.

Ophthalmology—Dr. Roman, of Springfield, chairman; Drs. Johnson, of Peoria, and Holmes, of Chicago.

Pulmonary Phthisis—Dr. S. W. Noble, of Bloomington, chairman; Drs. Law, of Dixon, and J. Adams Allen, of Chicago.

Otology—Prof. Isham, of Chicago, chairman; Drs. Winson, of Foreston, and I. F. Frazier, of Fayette county.

The following committees were appointed: On Intestinal Affections, Dr. I. D. Fitch, of Chicago; On the Duties of Society to the Profession, Dr. J. S. Whitmire, of Metamora; On Gleet, Dr. R. F. Higgins, of Vandalia; On Diabetes, Dr. W. D. Stirling, of Warren county; On Results in Fractures, Dr. L. W. Phillips, of Dixon; On Arrangements, Drs. Robert Baul, of Peoria, J. L. Hamilton, of Peoria, Roskoten, of Peoria, S. Maus, of Pekin, and Whitmire, of Metamora.

The following resolution was passed, on motion of Dr. Whitmire, of Metamora:

"*Resolved*, That it is the duty of the Legislature of the State to make such legal provisions as to insure persons who have committed capital crimes, and are acquitted of the same on the plea of insanity, being sent to the lunatic asylum as dangerous members of the community, and that a committee be appointed to procure such action."

The reading of the minutes was dispensed with till the evening session, and the convention proceeded to the regular business.

The following additional names as delegates and permanent members were reported: Professors E. Andrews and H. A. Johnson, of Mercy Hospital, Chicago, and Prof. E. Ingalls, of Rush Medical College, Chicago.

Dr. J. H. Hollister, Treasurer, then presented his annual report, which was substantially as follows:

| | |
|---|---|
| To balance in the treasury, May, 1869 | $ 86.00 |
| Dues paid by 60 of the members at annual meeting in Chicago | 240.00 |
| Received by mail from correspondence | 80.00 |
| Total | $406.00 |
| Paid out for publishing Transactions of the Society | 199.06 |
| Balance on hand | $206.94 |

Dr. Samuel J. Jones, of Chicago, then made a report on otology, which was, in substance, as follows: In view of the fact that no special report had ever been made on this subject, a cursory view would be given of its history. Instruments had been invented, within twenty-five years, which made the observations of the physician in diagnosis certain and reliable. Until recently, diseases of the ear had claimed but little interest or examination. Sir William Wylde, of Dublin, and Dr. Toynbee, of London, were the first who gave special and successful attention to aural diseases. Germany followed the example of England, and, with that patient research which characterizes her scientific men, she soon made gigantic advances in the science of otology. Inventive genius, when attention was turned to the subject, rapidly provided the lacking instruments. The Doctor here entered into a detailed description of the various instruments which were now in use among aurists, and also gave a list of the best and reliable works on the subject. There were two otological societies—the American and the German societies—which held regular annual meetings. The practical work accomplished had been very great. Many diseases, which formerly had baffled the physician, were now quite within his control.

Dr. Jones concluded with an elaborate and minute description of the principal diseases of the ear.

The report was referred to the committee on publication.

Prof. Holmes, of Chicago, presented a supplementary statistical report on otology. He said that this subject was left mostly to the practice of specialists. The time of preparation for general practice was so short, that few physicians were willing to give an opinion on severe and obscure diseases of the ear. About one-twelfth of the diseases treated by medicine were diseases of the eye, and the cases of diseases of the ear about one-fifth of those of the eye. During his twelve years of practice in Chicago, he had 9,006 cases of diseases of the eye, and 1,735 cases of diseases of the ear. Of the latter, 1,078 had been treated in hospital practice, and 678 in private. The number of cases under each special classification was given. Dr. Holmes closed his report with a warning to the profession against the use of the nasal douche in case of catarrhal affections of the ear, unless with great care. It should not be so far elevated as to cause a great pressure on the middle chamber of the ear, as thereby inflammation would be produced and the hearing seriously impaired.

Dr. Johnson, of Peoria, cautioned the profession against the use of the instrument known as the "eye sharpener," which has been extensively advertised as a remedy for near-sightedness. He had known cases where the lens of the eye was dislocated, and great extravasation of blood effected through this cause.

Several members of the convention also expressed their objections to the nasal douche on the same grounds as have already been mentioned, giving illustrations of the danger from their own practice.

The additional report of Dr. Holmes was referred to the committee on publication.

Prof. N. S. Davis, of Chicago, presented the preamble and resolutions adopted by the American Medical Association in May, 1869, as follows:

"*Whereas*, The history of medical legislation in the various States of this Union clearly shows that no reliance can be placed on either the uniformity or the permanency of any laws relating to the practice of medicine; and

"*Whereas*, The results of all the efforts made during the last twenty-five years to elevate the standard of medical education through concert of action among the numerous medical colleges of this country, have proved, with equal clearness, that such concert of action, in an efficient manner, is unattainable; therefore, be it

"*Resolved*, That whatever is done to establish and maintain a just and fair standard of medical education throughout our whole country must be done by the profession itself, through its own voluntary organizations, in the same manner that it now establishes and enforces its code of ethics. The profession is as competent to declare, through its representatives in the national, state and local societies, what shall be the standard of attainments for those to be recognized and admitted into its ranks, and to establish the boards or agencies by which compliance with such standard shall be ascertained, as it is to declare what shall be the ethical rules governing the conduct of those already admitted.

"*Resolved*, That this association earnestly request each state medical society to appoint annually one or more boards of examiners, composed of five thoroughly competent members, whose duty it shall be to meet, at suitable times and places, for the examination of all persons, whether graduates of colleges or not, who propose to enter upon the practice of medicine in their respective States, except such as have been previously examined and licensed by a similar board, in some other State.

"*Resolved*, That each state medical society be requested to require its examining board, or boards, to exact of every applicant, on examination, adequate proof that he has a proper general education; is twenty-one years of age, and has pursued the study of medicine three full years, one-half of which time shall have been in some regularly organized medical college, whose curriculum embraces adequate facilities for didactic, demonstrative, and hospital clinical instruction.

"*Resolved*, That each state medical society be requested to act on the foregoing propositions, at the next regular annual meeting after the reception of copies of the same, and if approved by the state medical societies of two-thirds of the states, this association shall deny representatives from all organizations who longer refuse to comply with the same, and shall recommend the state societies to do the same, and all persons who, after that date, seek to enter upon the practice of medicine without first receiving a license from the state board of examiners, shall be treated ethically as irregular practitioners.

"*Resolved*, That in adopting the foregoing resolutions, by which it is proposed to treat the medical college diploma the same as the diploma of any literary college, this association is actuated by no desire to injure the medical schools of our country. On the contrary, by the adoption of the fourth resolution at the same time that the value of the mere college diploma is practically nullified, it is the desire, and confident expectation, that those institutions will be greatly benefited; because they will be forced to rival each other in the extent and efficiency of their courses of instruction, instead of the number of diplomas which they can annually distribute.

Dr. Davis said the matter was one of great importance in the profession. The facts asserted in the preamble showed a great discord and want of harmony between the different colleges in various states, and that the state laws on the subject were entirely incomplete. Reform had been persistently urged by the American Medical Association in four several meetings. A uniform plan of medical education had been unanimously adopted by the American Medical Association, but it had remained a dead letter. The state of medical education in this country was exceptional, as no other profession contented itself with such fragmentary and imperfect *curricula* of study. Schools of medicine should be organized for instruction and culture exclusively, and not merely as rivals who were competing to attach the title of M. D. to as many as possible, without violating all the canons of educational respectability. Dr. Davis recommended most urgently that the state society should take official action sustaining the action of the American Medical Association. He proposed the following resolutions:

"*Resolved*, That this society cordially approves the propositions contained in the resolutions received from the American Medical Association.

"*Resolved*, That this society approves of the standard of preliminary education agreed upon by the convention of delegates for medical colleges in Cincinnati, in 1867, and approved by the convention in Washington, in May, 1870, except so much as relates to Greek and Latin; and that it is the duty of every state and local medical society to appoint annually a board of canons, whose duty it shall be to examine all who propose to enter upon the study of medicine in their respective districts, and to give a requisite certificate of qualifications; and further, where such board exists, it shall be regarded a violation of the ethics of the profession for any practitioner to receive a student into his office before he has received a satisfactory certificate from such board."

Prof. Ingalls, of Rush Medical College, opposed the passage of the resolutions at considerable length, on the ground that such union was impracticable and undesirable. The requirements of the different states demanded a different specialty of culture oftentimes. A firebrand would be introduced by the passage of these resolutions, that would cause infinite mischief; and he offered the following resolutions in place of the former:

"*Resolved*, That we acknowledge with gratitude and pride the advance the medical profession has made, and is continually making, in knowledge and scientific attainments, and that practical skill which enables its practitioners to combat disease and soothe the sufferings of the sick; and as far as we are able we will discard all personal feelings, and give our approbation and support to such colleges as impart the most thorough, extensive, and earnest teaching."

Dr. Young, of Aurora, attacked the action of the American Medical Association with great virulence, charging upon it that the members had been actuated by unworthy motives. He instanced the fact that colored members had been excluded from its deliberations. He thought that the reform could only be carried out by making a change in the fundamental law, and incorporating the provision in the state and national constitutions. To pass the proposed resolutions merely, would accomplish actually nothing.

Dr. Whitmire, of Metamora, thought the discussion was out of order, as the report of the committee was before the house for action. After remarks by Dr. Davis the question was called, and the report of Dr. Davis was received and referred to the committee for publication.

The committee on nominations made their report, and Peoria was appointed as the next place of meeting.

The following officers were elected for the ensuing year: President, Dr. G. W. Albin, of Neoga; First Vice-President, Dr. John Murphy, of Peoria; Second Vice-President, Dr. J. S. Whitmire, of Metamora; Permanent Secretary, Dr. T. D. Fitch, of Chicago; Assistant Secretary, Dr. J. R. Johnson, of Peoria; Treasurer, Dr. J. H. Hollister, of Chicago. The convention then adjourned till 7 o'clock P. M.

The afternoon was devoted to a delightful excursion and picnic, tendered by the citizens of Dixon, and held on the beautiful ground of Gov. Charters. The seat of Mr. Charters, located on a commanding bluff of the Rock river, about three miles from the town, was thrown open, both house and grounds, to the guests of the city, and a most generous hospitality lavished. A quadrille band was in attendance, and the afternoon was most agreeably spent in the various amusements in vogue on such occasions.

EVENING SESSION.

The evening session was called to order at 7 o'clock, Dr. Albin in the chair.

The following named gentlemen were proposed as permanent members: Drs. W. M. Maul, of Limerick, Bureau county, and J. K. Lewing, of Dover, Bureau county.

Prof. Ingalls, of Rush Medical College, made a brief volunteer report on scarlet fever. Scarlet fever was one of the most fatal in the list of diseases. More than 500 children in the last year, in Chicago, had perished under its ravages. The nature of the cause of scarlet fever he believed to be an organized poisonous germ of some sort not fully known, but resulting in a process of fermentation in the blood. Nothing but an organism would produce its like. This was the case with scarlet fever, so it must be the result of the same living germ. Sometimes atmospheric causes might produce it spontaneously, but in the majority of cases it was by infection. Having thus originated once, the disease was infinitely reproduced. The severity of the reproduction depended on the character of the original germ. In some cases it was severe and virulent, while sometimes mild. As to prevention, the problem had been greatly overlooked by physicians. Many men were ignorant of the fact that scarlet fever was contagious. This ignorance was unpardonable. Much of the extent of the disease was due to this ignorance. Every case should quarantined. It was better and easier to prevent the disease than to cure it. Another means of prevention was ventilation, which diluted the germ, and prevented, to a large extent, its fatal effects. Another means was that of disinfection—an agency not fully appreciated by medical men. The discharge, the air, and the clothing should be thoroughly disinfected. He briefly discussed the cure of the disease. All that was claimed for the cure of scarlet fever was the conduct of it to a safe issue. It could not be checked prematurely. He had no confidence in any special medicine. Oxygen set free in the blood was the best. He preferred chlorate of potash, as modifying the course of the disease. He did not believe in mercurials in any shape. They did positive injury. Cases should not be much purged. If laxatives were used, they should be of the most gentle nature. He would not elaborate much now in regard to the remedies, but would give a full expression of his views in his formal written report for publication.

Dr. Young, of Aurora, differed from Prof. Ingalls. He believed in an active, heroic treatment of this disease.

Prof. Davis thought, as the subject had been discussed, the paper of Dr. Ingalls should be referred to the committee on publication. The motion was carried.

Prof. Powell, of Chicago, gave a brief report of a case of amputation of the hip. He detailed the history of the patient as relating to the disease. The case was treated by perfect rest and warm fomentations of the knee and hip during the incipient stage. An injury received by a fall aggravated the disease, and finally amputation became necessary. When the operation was performed, the patient was nearly skin and bone, emaciated to the last degree. After the operation was performed, the patient was so exhausted that he only breathed four times a minute. That patient was still living, and was now in a fair way of convalescence in the hospital. The point of interest was the propriety of performing the operation of amputation at the hip-joint in case of caries of the bone. The report was referred to the committee on publication.

The committee on nominations made a further report, as follows: Committee on Criminal Insanity, Drs. A. McFarland, of Jacksonville, J. S. Whitmire, of Metamora, and R. J. Patterson, of Jacksonville; Idiocy, Drs. C. K. Wilbur, of Jacksonville, J. R. Eillane, of Jacksonville, and Harrison Noble, of Heyworth; the Medical Use of Carbolic Acid, Drs. D. C. Law, of Dixon, and John Murphy, of Peoria; Cholera Infantum, Dr. D. W. Young, of Aurora; Membranous Croup, Dr. David Prince, of Jacksonville.

The following were appointed as delegates to the medical societies of adjoining States: Iowa, S. C. Plumba, W. D. Sterling; Missouri, P. Cook, S. P. Breed; Indiana, W. H. Byford, J. P. Ross; Wisconsin, W. C. Lyman, D. C. Foot; Kansas, G. W. Phillips, J. M. Moore, J. S. Whitmire; Ohio, Dr. Chas. Hunt.

The following members were elected as delegates to the National Association: Drs. N. S. Davis, of Chicago; J. O. Hamilton, of Jerseyville; A. W. Young, of Aurora; S. J. Jones, of Chicago; E. Ingalls, of Chicago; R. J. Higgins, of Vandalia; —— Johnson, of Peoria; E. Powell, of Chicago; R. Isham, of Chicago; W. W. Winn, of Dixon; H. A. Johnson, of Chicago; G. J. Monroe, of Leland; J. S. Whitmire, of Metamora; Moses Gunn, of Chicago; D. J. Crist, of Bloomington; M. M. Robbins, of Aurora; M. W. Walton, of Ridout; C. G. Amity, of Chicago; J. M. Morse, of Galesburg; J. M. Ainsley, of Galesburg; J. P. McClanahan, of Galesburg; G. W. Heath, of Franklin Grove; and E. P. Cook, of Mendota.

On motion of Dr. Young, the thanks of the society were voted to the mayor, common council, and citizens of Dixon, for the magnificent reception accorded them.

The resolutions offered by Prof. Ingalls in the morning were, by a vote of the society, appended to the report of Prof. Davis.

On motion of D. P. Cook, of Mendota, it was resolved that a committee should be appointed to urge upon the legislature the necessity of an official registration of births, marriages, and deaths, for the State, and that Dr. Rauch, of Chicago, should be the chairman of the committee. Dr. David Prince, of Jacksonville, and H. A. Johnson, of Chicago, were appointed as his associates.

On motion of Dr. Samuel Jones, of Chicago, it was voted that a unanimous vote of thanks should be given to the presiding officer of the society for the able manner in which he had discharged his duties.

Dr. Albin made a short speech, acknowledging the kindly feelings of the society. The society then adjourned till the third Tuesday in May of 1871.

